# IPA Analysis of Cervicovaginal Fluid from Precancerous Women Points to the Presence of Biomarkers for the Precancerous State of Cervical Carcinoma

**DOI:** 10.3390/proteomes2030426

**Published:** 2014-08-13

**Authors:** Xaveer Van Ostade, Martin Dom, Geert Van Raemdonck

**Affiliations:** 1 Laboratory for Protein Science, Proteomics and Epigenetic Signaling (PPES), University of Antwerp, Universiteitsplein 1, Wilrijk 2610, Belgium; E-Mails: Martin.Dom@uantwerpen.be (M.D.); Geert.VanRaemdonck@uantwerpen.be (G.V.R.); 2 Centre for Proteomics and Mass Spectrometry (CeProMa), University of Antwerp, Universiteitsplein 1, Wilrijk 2610, Belgium

**Keywords:** cervicovaginal fluid, biomarker, cervical cancer

## Abstract

Despite large gaps in our knowledge on the intracellular mechanism leading to cervical cancer, the pathways induced by oncogenic high-risk Human Papilloma Virus (HPV) and those finally causing cervical cancer are increasingly being unraveled. Assuming that precancerous tissue is recognized and lysed by the immune system—which is in many cases incomplete because of the counteraction by the HPV virus—we hypothesize that several intracellular factors, involved in induction and development of precancerous lesions and/or cervical cancer are being released into the cervicovaginal fluid (CVF). These factors can then be seen as markers for the precancerous state, and when they persist they are indicative for an increased risk for cervical carcinoma. In a previous study, we analyzed the proteomic profiles of six CVF samples from women with different stages of precancerous lesions and compared these with the CVF proteomes from healthy women. Here, we extend these observations by investigating these proteomes by Ingenuity Pathway Analysis (IPA). We show that proteins in CVF from precancerous women are clearly more involved in pathways that make up the ‘hallmarks of cancer’, as compared to CVF proteins from healthy persons. Moreover, after literature search, proteins classified by IPA in the ‘cancer’ category, were more correlated with cervical cancer when they originated from CVF from precancerous women. Many of these proteins formed a network with angiotensin II as central mediator. The search for ‘network biomarkers’, rather than single biomarkers, could drastically increase specificity, sensitivity and prognostic value of cervical cancer diagnosis, making use of an easy to handle fluid, the CVF.

## 1. Introduction

### 1.1. Cervicovaginal Fluid (CVF): An Underestimated Source of Biomarkers for Pathologies of the Female Genital Tract

If we consider an organismal secretion as a substance that is produced and transported outside an organism, then we can conclude that thanks to organismal secretions, every living organism, daily, leaves numerous fingerprints behind, and humans, with their complex physiology, are certainly not an exception to this rule. Indeed, humans produce a wide array of different secretions according to the different organs and tissues that come into contact with the exterior. Many secretions have a specific physiological function and act as physical and chemical barriers against pathogens that may come into contact with the soft, non-keratinized tissue separating the organ from the exterior. Moreover, they often serve to protect the organ from waste products that are the result of metabolic and physiological processes. One could, therefore, consider organismal secretions as relatively easy obtainable fingerprints, handed to us by nature, as to inform us about the physiological state of the inside of our body and its state of defense against assaults from the outside. Examples of such secretions are feces, urine, brochoalveolar fluid, sweat, milk, tear fluid, saliva, semen, or cervicovaginal fluid.

The above consideration means that the cervicovaginal fluid (CVF) may contain a wealth of components informing us about the condition of many organs of the female reproductive system. This fluid includes secretions from many sources, such as vulvar secretions, plasma transudate, exfoliated cells, cervical mucus, endometrial and oviductal fluids, and secretions from vaginal immune cells [1,2]. In addition, CVF also contains commensal aerobic and anaerobic microorganisms and their corresponding products [3,4]. As a result, this fluid comprises a wide array of components ranging from inorganic ions over lipids, carbohydrates and proteins to immune cells and microorganisms. It is believed that in a healthy situation these factors are in equilibrium although this equilibrium can be modulated by processes such as menstruation or pregnancy. However, if a pathology develops at some part in the female genital tract, the balance will be disturbed at two time points, thereby changing the abundance of one or more of the CVF components and thus leaving a fingerprint for the disease. At first, deregulated cells may secrete small amounts of unusual components into the CVF. It is expected that the proportion of these molecules into the CVF will very much depend on the nature and the extent of the pathology and, in many cases, levels of these signal molecules will be below detection levels of current diagnostic methods. However, given the role of CVF in the innate and adaptive immune response, and the very large array of molecules immune cells can secrete [5,6], indirect immune biomarkers may arise at a later time point and at a much higher concentration as compared to the original trigger molecule(s). However, to extract from an immune response the nature of a disease is still a huge challenge and, thus, it is much more efficient to trace the molecules from the target that are released in the CVF following the attack by the immune system.

A main advantage of organismal secretions is the ease of collection as in most cases no invasive collection method is required. In the case of CVF, lavages or methods using swabs or tampons can be applied by the practitioner or even by the woman herself [7,8]. CVF is, therefore, very well suitable for regular diagnosis and follow-up of gynecological diseases, especially in regions where medical practitioners are difficult to reach. Moreover, in contrast to, for instance, serum, the volume of CVF is milliliter quantities, hence, the biomarker will be present in a high concentration thereby avoiding the need for concentration of low abundance biomarkers before detection. Although plasma components are present in CVF as a result of transudation, only organs of the female genital tract (vagina, cervix, uterus, endometrium, Fallopian tubes and ovaries) secrete their products into the CVF so that the ratio of these gynecological biomarkers over plasma components is high and drastically increases the specificity of CVF for malignancies of the female genital tract.

### 1.2. Cancer and the Necessity for Detecting Several Biomarkers Simultaneously

Development of a cancerous state in the cell is now considered to be a distortion of several intracellular pathways, repeatedly observed in many different neoplastic cell types and thus representing key processes for cancer to develop. These so called hallmarks of cancer encompass several biological features that gradually accumulate during development of neoplastic tissue: continuous growth signaling, suppression of growth inhibitors, resisting apoptosis, enabling immortality by promoting replication, angiogenesis induction, enhanced invasion and metastasis, adaption of energy metabolism, and escaping the immune response [9,10]. Although these hallmarks are an excellent tool to intellectually tackle the complexity of tumor genesis, the modes by which tumors acquire these features vary considerably and are still the main subject of current molecular cancer research. This diversification of cancer etiology and development renders every type of tumor its characteristic overabundant components and the challenge for cancer biomarker research is to detect these components. Only determination of the correct set of biomarkers will unambiguously identify early presence of the tumor, tumor type, and, if possible, its development state. Undoubtedly, proteins make up a large part of this characteristic set.

### 1.3. How Cervical Carcinoma Cells may Deliver Their Cancer Biomarkers into the CVF

Tumor cells can express characteristic membrane molecules allowing detection by several biochemical assays. However, in the case where body fluids are used for cancer diagnosis, a considerable number of tumor cells must be presenting the body fluid to make detection by the bioassay possible and enrichment of circulating tumor cells is often necessary [11,12,13]. This is not the case with cervical cancer, where tumor cells need to be scraped off the cervical epithelial lining by swabs in order to be detectable by bioassays, such as those used for cytology [14]. The question therefore remains whether, and, if so, how these tumor cells secrete protein biomarkers into the CVF. As HPV infection of the basal or suprabasal layer cells of the cervical epithelium and subsequent productive proliferation of the virus at the upper epithelial layers does not result in necrosis nor apoptosis of the infected cells, release of HPV-related proteins into the surroundings does not occur and an anti-HPV immune response is avoided [15]. Moreover, these virus producing cells are not tumorigenic, a capacity only attributed to the infected basal membrane cells that develop via the CIN1-3 precancerous states to a metastatic cervix carcinoma [16]. During this period that usually takes several years, immune surveillance against precancerous tissue may still be functional, causing the lysis of the intraepithelial lesion, despite counteraction by HPV [17]. Indeed, although HPV E6 and E7 oncoproteins directly interact with components of the interferon signaling pathways [18] and, therefore, alter the expression of genes (interferon response genes, NF kappa B stimulated genes, and cell cycle regulation genes) that enable host resistance to infection and immune function [19,20], in 80% of the cases CIN1 or 2 lesions are cleared [21], pointing to the existence of an immune system that is at least partially active.

We, therefore, speculate that the crippled immune response towards intraepithelial lesions may still be effective enough to result in a degree of cell lysis that provides us with enough quantities of fingerprint molecules for detection by for example antibody- or mass spectrometry-based assays.

In our previous paper [22], a differential shotgun proteomics strategy showed the regular appearance of six proteins in the CVF from precancerous women, while none of them was observed in CVF from healthy women. Although this qualitative difference led to the identification of at least one potential biomarker for the precancerous state of cervix cancer, we assumed that, due to sensitivity limitations of the shotgun proteomics, many biomarkers that were scarcely and/or not regularly detected could be measured and quantified in a more sensitive targeted method, such as the enzyme-linked immunosorbent assay (ELISA) or Multiple Reaction Monitoring (MRM) mass spectrometry. The aim of this study is therefore to reconstruct on the basis of the fragmented information from the two lists of Van Raemdonck *et al*. [22] (proteins unique to ‘healthy’ and ‘precancerous’ samples), the pathways involved in cervical cancer and their proteins that ‘leak’ into the cervicovaginal fluid. To this end, we will use CVF proteins that show a qualitative difference (*i*.*e*., unique to one of the two conditions) and, although these often only appear in one or a few samples from one condition, their clustering into a limited number of pathways may point to their relevance, especially when this clustering would be more efficient with CVF proteins from precancerous women. This may provide us with hints for other biomarkers (and combinations thereof) to detect in sensitive targeted assays such as ELISA or MRM.

## 2. Experimental

### 2.1. Study Design and Sample Collection

All patients agreed to participate by written consent and the study was approved by the ethical committee of the Antwerp university hospital (Registration Number: B30020108372). Collection of samples was described previously [22]. In short, samples came from women who visited the University Hospital of Antwerp because of abnormal Pap smear results. All patients were routinely subjected to a colposcopic examination, a procedure that includes rinsing the vagina with 5% acetic acid. This washing fluid (containing the cervicovaginal fluid) is normally discarded but was collected for proteomic analysis. In addition, cervical cytology samples were collected to determine the cytology and HPV status by type-specific PCR. In some cases, no precancerous tissue and high-risk HPV (HR-HPV) infection could be observed. These women were classified as ‘healthy’, since it is well known that Pap smears often result in false positives [23].

We selected samples, originating from six healthy (normal colposcopy/cytology and HPV negative) and six precancerous (abnormal colposcopy/cytology and HR-HPV positive) individuals. All samples were derived from postmenopausal (> three years after the last menses) women from similar age (59 ± 13 years). These women did not use hormone replacement therapy and were free of bacterial vaginosis (Table 1).

**Table 1:** Patient information of the six healthy and six precancerous samples used for differential proteomics in Van Raemdonck *et al*. [22].

Sample	Age	Genotype(s)	Viral load	Colposcopy	Cytology
A.1	57	no HPV		Normal	Normal
A.2	69	no HPV		Normal	Normal
A.3	52	no HPV		Normal	Normal
A.4	46	no HPV		Normal	Normal
A.5	66	no HPV		Normal	Normal
A.6	62	no HPV		Normal	Normal
B.1	61	31/52	335/2	LSIL	CIN1
B.2	53	16	417	HSIL	CIN3
B.3	47	39	2011	LSIL	CIN2
B.4	72	18/56	0.1322/1119	LSIL	CIN1
B.5	57	31	1148	HSIL	CIN3
B.6	60	31/52	258/25	LSIL	CIN2

### 2.2. Proteomic Analysis

Proteomic analysis was previously described in more detail [22]. Cervicovaginal lavages (25–40 mL) were immediately transported on ice to the laboratory and stored at −80 °C. For analysis, samples were thawed, centrifuged, and supernatant was concentrated by lyophilisation to a final volume of approximately 200 µL. An amount of 1 mg of proteins was first separated and fractionated on a reverse phase (RP) protein C4 HPLC column. Fractions were digested with trypsin and resulting peptides of each fraction were separated in a second dimension on a RP*-*C18 micro-capillary HPLC system. Mass spectrometric analysis was performed using a MALDI-ToF/ToF. Resulting MS/MS Spectra from each sample were screened against the human Swiss-Prot database (version: 57.1) using the MASCOT search engine. Analysis of the obtained datasets was performed as previously described [24].

### 2.3. IPA Analysis

CVF proteins from the six healthy and six precancerous women were pooled in two datasets (‘healthy’ and ‘precancerous’) and the gene/protein ID numbers from each list were uploaded to the Ingenuity Pathway Analysis (IPA) software [25]. IPA uses a knowledgebase derived from the scientific literature to relate genes or proteins based on their interactions and functions. Based on the uploaded dataset, the program algorithmically generates biological networks and defines canonical pathways and functions. Individual networks receive a score that is derived from a *p-*value (score = −log (*p-*value)) indicating the likelihood that focus proteins (*i*.*e*., the identified proteins within a network) are clustered together. A right-tailed Fisher’s exact test is used for calculating the *p-*values. The *p-*value determines the probability by which association between the proteins in the dataset and the functional and canonical pathway could happen by chance alone. The final scores are expressed as negative log of *p-*values or by *p-*values and used for ranking. *p-*Values of less than 0.05 were considered significant. IPA also calculates a ratio, which indicates the strength of association with a canonical pathway. From these two numbers, IPA determines the most significant canonical pathways associated with the dataset.

## 3. Results

### 3.1. IPA Analysis of CVF from Healthy Individuals versus CVF from Individuals in the Precancerous State: Rationale

A proteomics platform, consisting of two-dimensional Liquid Chromatography (2D-LC), coupled to a Matrix-Assisted Laser Desorption Ionization tandem Time-off-Flight Mass Spectrometer (MALDI-TOF-TOF) has been shown to be very efficient for identification of cervicovaginal proteins [24]. Using this platform, we performed a differential proteomics experiment on six individual CVF samples, originating from healthy persons *versus* six individual samples from persons with low grade squamous intraepithelial lesions (LSIL) or high grade squamous intraepithelial lesions (HSIL) (“precancerous individuals”). We assumed that precancerous tissue probably has already several hallmarks of cancer acquired, making it vulnerable to immune action. This would result in partial cell lysis and subsequent release of proteins, including overexpressed proteins that are involved in cervical cancer. The results from our study led to the identification of a potential CVF biomarker for the precancerous state of cervical cancer and were published elsewhere [22]. Additional candidate biomarkers were observed but could not be determined with statistical relevance due to the small number of samples (2 × 6). We, therefore, looked for biological evidence for those proteins that occurred in the CVF of (some) individuals with LSIL or HSIL lesions, but not in the CVF of healthy individuals. Since it can be expected that overexpressed proteins in cancer cells often belong to a set of cancer hallmark pathways, many of these proteins will be interconnected. As some of these proteins leak into the CVF, their interconnection and presence in cancer pathways is, therefore, a strong indication for correlation with cervical cancer and its precancerous state. We, therefore, reasoned that, instead of single protein biomarkers, pathway biomarkers may bring about an increased specificity and sensitivity in diagnosis. We, therefore, further investigated the lists of identified proteins by the highly curated software package IPA [25] in order to further select for ‘pathway biomarkers’ that correlate with the precancerous state of cervical cancer.

### 3.2. Predicted Pathways Formed by CVF Proteins from Healthy or Precancerous Women Have a Similar Functional Distribution but Differ in Confidence

The CVF proteomes from six healthy and six precancerous women were identified, resulting in two lists of 371 and 341 proteins, respectively. As 238 of these proteins are overlapping, CVF proteomes from healthy and precancerous women had 133 and 103 unique proteins, respectively (for the complete lists: see Van Raemdonck *et al*. [22]). Both lists were further investigated using IPA for calculation of the degree to which proteins within one list are interconnected.

The *p-*value ascribed to a pathway in IPA represents the likelihood that the association between a given set of proteins and a pathway is due to random chance. Hence, the smaller the *p-*value, the more significant the association is. The *p-*value is dependent on the number of proteins within the set and the total number of proteins, known to be associated with that pathway. The more proteins involved, the more likely the association is not due to random chance, which is reflected in a lower *p-*value.

**Figure 1:**
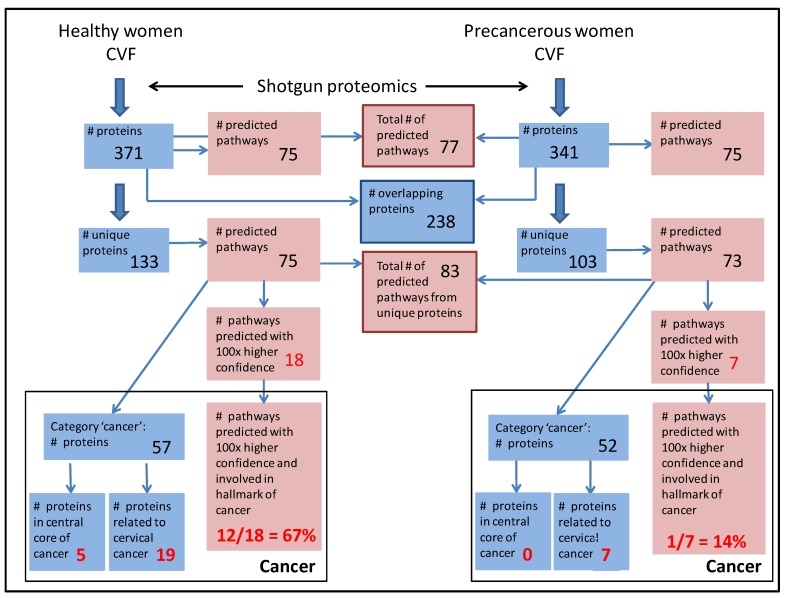
Overview of IPA analyses on CVF proteomes from precancerous (**right**) and healthy (**left**) women. Protein and pathway features are depicted in blue and pink, respectively. Common features are framed in the same color. Numbers of proteins and within pathway features with more than 50% difference between the precancerous and healthy state are shown in red. All features that concern cancer are grouped and framed in black.

**Figure 2:**
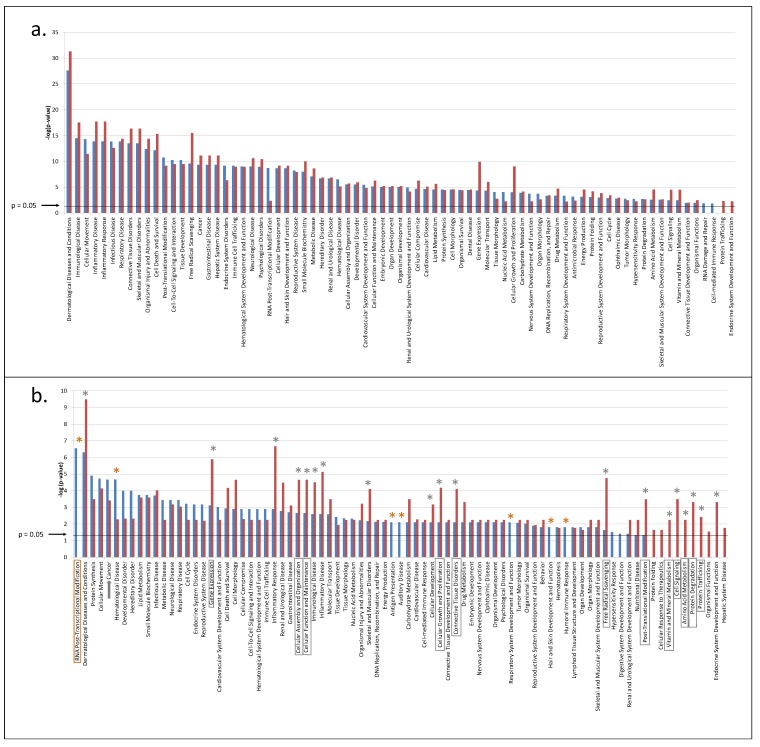
(**a**) IPA pathways, assembled with high confidence (*p <* 0.05) from all CVF proteins originating from healthy (blue bars) and precancerous (red bars) women; (**b**) IPA pathways, assembled with high confidence (*p <* 0.05) from proteins that are unique in CVF from healthy (blue bars) and precancerous (red bars) women. Pathways, showing a marked difference in *p-*value (>10^2^) between both conditions are marked with a brown (>10^2^ more confident in samples from healthy persons) or grey (>10^2^ more confident in samples from precancerous persons) asterisk. From these, pathways belonging to the hallmarks of cancer are framed in according colors. Assembly of cancer pathways is marked by a grey arrow. Details can be found in Supplementary Files 1 and 2.

Seventy-seven statistical relevant pathways were predicted, involved in a wide array of biological functions and with *p-*values ranging from 4.83 × 10^−32^ (Dermatological diseases and conditions) till 5.32 × 10^−3^ (Endocrine system development and function) (Figure 1 and Figure 2a, Supplementary File 1). Although nearly all of the pathways were present in CVF samples from both types of individuals, the majority was present with distinct higher confidence (hence containing more interconnected proteins) in CVF from precancerous women. Only three pathways were predicted with a clearly higher confidence for samples from healthy women (Figure 2a, Supplementary File 1). These were involved in ‘Post transcriptional modification’ and to a lesser extent ‘RNA damage and repair’ and ‘Cell mediated immune response’. While it is not yet clear why the first two pathways are more absent in CVF from precancerous patients, the absence of CVF proteins involved in cell mediated immunity could be explained by the capacity of HPV to suppress several aspects of the cell-mediated immune response (see above).

### 3.3. CVF from Precancerous Patients Contains Substantially More Unique Proteins That Are with Higher Confidence Involved in Pathways, Related to the Hallmarks of Cancer

To further investigate the above findings, we looked at the confidence of pathway assembly using proteins that were unique in CVF from precancerous *versus* healthy women. If only these proteins were considered, 83 pathways could be assembled with high confidence (*p <* 0.05) (Figure 1 and Figure 2b, Supplementary File 2). Of note, the reason for the increase from 77 to 83 pathways lies in the higher stringency the program uses when a larger input data set is given. Analogous to above findings, it was clear that in CVF samples from precancerous women more pathways could be assembled to a clearly higher degree of confidence (*p-*value at least 100-fold lower) as compared to CVF samples from healthy women: respectively 18 *versus* 7. More important, in samples from precancerous women, a substantial part of these pathways (12/18 (67%)) is linked to processes, related to the hallmarks of cancer (compare with CVF samples from healthy women: 1/7 (14%)) (Figure 1 and Figure 2b, Supplementary File 2).

#### 3.3.1. CVF from Precancerous Patients Contains Substantially More ‘Cancer Proteins’ That Are Involved in Cervical Cancer and That Are Interconnected

**Figure 3:**
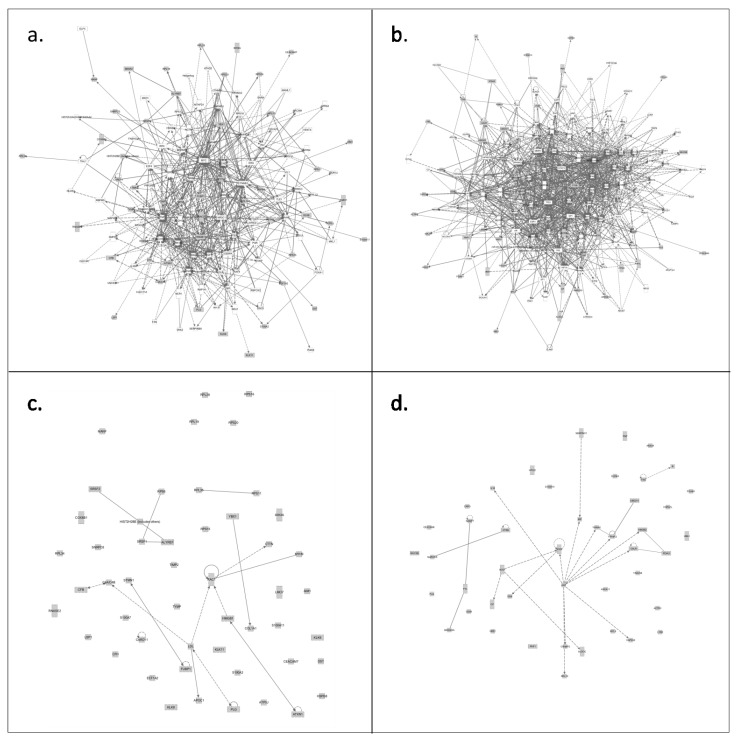
IPA constructed pathways from (**a**) interconnecting unique CVF proteins from healthy individuals and their first neighbors (**b**) interconnecting unique CVF proteins from precancerous individuals and their first neighbors. Removing the first neighbors and their connections resulted in respectively (**c**) and (**d**). Full and hatched lines represent direct and indirect interactions, respectively. Details can be found in Supplementary Files 3 and 4.

Above findings suggested that more interconnected proteins, involved in cancer processes, were present in CVF from precancerous women. Unexpectedly, assembly of ‘cancer’ pathways was somewhat better using unique CVF proteins from healthy persons (*p =* 2.16 × 10^−5^) compared to those from precancerous persons (*p =* 4.95 × 10^−4^) (Figure 2b, Supplementary File 2) and only a slight increase in unique proteins falling under the IPA category ‘cancer’ was found in CVF from precancerous women (57 (precancerous) *versus* 52 (healthy), Figure 1; Table 2). The reason for this result may lie in the unavoidable limitations for annotation of the ‘cancer pathway’. Indeed, many proteins are not recorded as cancer proteins although their involvement in a cancer hallmark pathway is their contribution to the cancer process. Nevertheless, we manually investigated the literature of those unique proteins in CVF from healthy and precancerous individuals, falling under the class of ‘cancer pathway proteins’ (Table 2). For this, we used the key word combination “XXX_HUMAN” (XXX = name of the protein, according to UniProt nomenclature) and “cervical cancer” as to find out whether relations between these proteins and cervical cancer have been described in literature. In addition, several high-throughput differential proteomics studies on cervical cancer [26,27,28,29,30,31] were screened for differential abundance of these proteins. Results showed that a markedly higher number of unique CVF proteins from precancerous women were described to be involved in cervical cancer: 19 (precancerous) *versus* 8 (healthy). For each of these 19 proteins, a short description of this relationship is summarized in Table 3. The substantially higher number of cervical cancer-related proteins in CVF from precancerous individuals suggests that CVF from these patients may contain more biomarkers that correlate with cervical cancer as previously expected. Moreover, from these 19 proteins, 12 were involved in a network wherein angiotensin II, an enzymatic product of angiotensinogen, had a high degree of indirect centrality (Figure 1 and Figure 3c,d, Supplementary File 3), and extension of the networks with first neighbor proteins resulted in a denser network among CVF proteins from precancerous individuals as compared to those from healthy women (Figure 1 and Figure 3a,b, Supplementary File 4).

**Table 2:** List of unique CVF proteins from precancerous (**left**) and healthy (**right**) individuals, allowing assembly of cancer pathways by IPA with a high degree of confidence (*p <* 0.05). Proteins described to be involved in cervical cancer are marked in grey.

Precancerous-Category Cancer		Healthy-Category Cancer		
Protein Name	Protein ID	Protein Name		Protein ID
14-3-3 protein epsilon	1433E_HUMAN	40S ribosomal protein S11		RS11_HUMAN
14-3-3 protein theta	1433T_HUMAN	40S ribosomal protein S15		RS15_HUMAN
15-hydroxyprostaglandin dehydrogenase [NAD(+)]	PGDH_HUMAN	40S ribosomal protein S16		Q6IPX4_HUMAN
60S ribosomal protein L15	RL15_HUMAN	40S ribosomal protein S20		RS20_HUMAN
Actin-related protein 3	ARP3_HUMAN	40S ribosomal protein S6		RS6_HUMAN
Angiotensinogen	ANGT_HUMAN	60S ribosomal protein L19		RL19_HUMAN
Annexin A11	ANX11_HUMAN	60S ribosomal protein L28		RL28_HUMAN
Annexin A4	ANXA4_HUMAN	60S ribosomal protein L34		RL34_HUMAN
Antithrombin-III	ANT3_HUMAN	60S ribosomal protein L35		RL35_HUMAN
Argininosuccinate synthase	ASSY_HUMAN	60S ribosomal protein L7		RL7_HUMAN
Calreticulin	CALR_HUMAN	Apolipoprotein C-1		APOC1_HUMAN
Carcinoembryonic antigen-related cell adhesion molecule 6	CEAM6_HUMAN	Ataxin-1-like		ATX1_HUMAN
Cathepsin B	CATB_HUMAN	ATP synthase-coupling factor 6, mitochondrial		ATP5J_HUMAN
CD59 glycoprotein	CD59_HUMAN	Carcinoembryonic antigen-related cell adhesion molecule 7		CEAM7_HUMAN
Cellular retinoic acid-binding protein 2	RABP2_HUMAN	Caspase recruitment domain-containing protein 11		CAR11_HUMAN
Centriolin	CNTRL_HUMAN	Collagen alpha-1 (I) chain		CO1A1_HUMAN
Ceruloplasmin	CERU_HUMAN	Complement C4A/C4B		CO4A_HUMAN, CO4B_HUMAN
Copine-7	CPNE7_HUMAN	Complement factor B		CFAB_HUMAN
Cytosolic non-specific dipeptidase	CNDP2_HUMAN	Complement factor H		CFAH_HUMAN
F-actin-capping protein subunit alpha-2	CAZA2_HUMAN	Cytochrome c oxidase subunit 6B1		CX6B1_HUMAN
Ferritin light chain	FRIL_HUMAN	Dystonin		DYST_HUMAN
Filaggrin	FILA_HUMAN	Elongation factor 1-alpha 2		EF1A2_HUMAN
Fructose-biphosphate aldolase C	ALDOC_HUMAN	Far upstream element binding protein 1		FUBP1_HUMAN
Gelsolin	GELS_HUMAN	Heat shoch protein beta-8		HSPB8_HUMAN
Guanylate-binding protein 6	GBP6_HUMAN	High mobility group protein B1		HMGB1_HUMAN
Heat shock 70 Da protein 1-like	HS71L_HUMAN	Histone H2B type 2-E		H2B2E_HUMAN
Hemoglobin subunit epsilon	HBE_HUMAN	Isocitrate dehydrogenase [NAD] subunit alpha, mitochondrial		IDH3A_HUMAN
High mobility group protein B2	HMGB2_HUMAN	Kalikrein-11		KLK11_HUMAN
Inter-alpha-trypsin inhibitor heavy chain H2	ITIH2_HUMAN	Kallikrein-6		KLK6_HUMAN
Interleukin-18	IL18_HUMAN	Kallikrein-8		KLK8_HUMAN
Junction Plakoglobin	PLAK_HUMAN	LIM domain only protein 7		LMO7_HUMAN
Lyphocyte antigen 6D	LY6D_HUMAN	Lymphocyte-specific protein 1		LSP1_HUMAN
Macrophage migration inhibitory factor	MIF_HUMAN	Membrane-associated transporter protein		S45A2_HUMAN
Macrophage-capping protein	CAPG_HUMAN	Metalloproteinase inhibitor 2		TIMP2_HUMAN
Mucin-5B	MUC5B_HUMAN	Mesencephalic astrocyte-derived neurotrophic factor		MANF_HUMAN
Myosin light polypeptide 6	MYL6_HUMAN	Myosin-9		MYH9_HUMAN
Myristoylated alanine-rich C-kinase substrate	MARCS_HUMAN	Non-secretory ribonuclease		RNAS2_HUMAN
Nicotinamide phosphoribosyltransferase	NAMPT_HUMAN	Nuclease-sensitive element binding protein 1		YBOX1_HUMAN
Olfactomedin-4	OLFM4_HUMAN	Plasminogen		PLMN_HUMAN
PHD finger protein	PHF1_HUMAN	Protein disulfide isomerase A6		PDIA6_HUMAN
Phosphoglycerate mutase 1	PGAM1_HUMAN	Protein S100-A11		S10AB_HUMAN
Protein disulfide isomerase A3	PDIA3_HUMAN	Protein S100-A2		S10A2_HUMAN
Protein S100-A14	S10AE_HUMAN	Protein S100-A7		S10A7_HUMAN
Protein S100-P	S100P_HUMAN	Ras-related C3 botulinum toxin substrate 1		RAC1_HUMAN
Purine nucleoside phosphorylase	PNPH_HUMAN	Serine/arginine-rich splicing factor 1		SRSF1_HUMAN
Puromycin-sensitive aminopeptidase	PSA_HUMAN	Small nuclear ribonucleoprotein Sm D3		SMD3_HUMAN
Putative hydroxypyruvate isomerase	HYI_HUMAN	Src substrate cortactin		SRC8_HUMAN
Rho GD*P-*dissociation inhibitor 1	GDIR1_HUMAN	Stathmin		STMN1_HUMAN
Serpin B12	SPB12_HUMAN	THO complex subunit 4		THOC4_HUMAN
Serpin B13	SPB13_HUMAN	Thymidine phosphorylase		TYPH_HUMAN
Sororin	CDCA5_HUMAN		Voltage-dependent P/Q-type calcium channel subunit-1alpha	CAC1A_HUMAN
Superoxide dismutase [Cu-Zn]	SODC_HUMAN		Zink-finger and BTB domain-containing protein 4	ZBTB4_HUMAN
Superoxide dismutase[Mn]	SODM_HUMAN			
Transgelin-2	TAGL2_HUMAN			
Transmembrane protease serine 11B	TM11B_HUMAN			
Ubiquitin-conjugating enzyme E2 variant 1	UB2V1_HUMAN			
Ubiquitin-like modifier-activating enzyme 1	UBA1_HUMAN			

**Table 3:** List of cervicovaginal fluid proteins, unique for precancerous patients, allowing assembly of cancer pathways by Ingenuity Pathway Analysis and described to be involved in cervical cancer to some extent. For 14-3-3θ and ε, involvement in cervical cancer was decided on the basis of the ζ and σ isoforms. SCC = squamous cell carcinoma.

No.	Gene Name	Protein ID	Protein Name	Cell Line, Tissue or Patient	Effect	Ref.
1.	AGT	ANGT_HUMAN	Angiotensinogen	IHC was used for investigation of angiotensin receptor levels in normal and neoplastic cervical tissues and Siha cells. Invasion assay was examined in Siha cells and vascular endothelial growth factor levels were assayed by ELISA.	Precursor of Angiotensin II. Ang II is involved in the progression of cervical carcinoma via induction of VEGF secretion through angiotensin II type I receptor. This results in increased invasiveness of carcinoma cells. Correlation of angiotensin II type I R expression with progression from precancerous to invasive cervical carcinoma.	[32]
2.	ANXA4	ANXA4_HUMAN	Annexin A4	Differential proteomics of HeLa cells *versus* non-tumorigenic cell line HaCaT	Annexin A4 is overexpressed in HeLa cells as compared to HaCaT	[26]
3.	CAPG	CAPG_HUMAN	Macrophage-capping protein	Differential proteomics of HeLa cells *versus* non-tumorigenic cell line HaCaT	CAPG is overexpressed in HeLa cells as compared to HaCaT	[26]
4.	CD59	CD59_HUMAN	CD59 glycoprotein	CD59 expression was examined in normal, precancerous and cervical squamous carcinomas samples.	CD 59 is abundantly present in cervical carcinomas although staining of tumor cells appears less intense than staining of adjacent stroma.	[33]
				IHC of CD59 was performed on cervical carcinomas, normal cervical epithelial cells, and the surrounding stroma.	CD59 expression was shown on cervical carcinomas, normal cervical epithelial cells, and the surrounding stromal cells. CD59 was a potent inhibitor of classical pathway-mediated lysis on cervical cancer cell lines.	[34]
5.	CTSB	CATB_HUMAN	Cathepsin B	Investigation of mRNA expression profiles of 8 thermoradiosensitive and 11 thermoradioresistant cervical tumors obtained by punch biopsy before treatment using a cDNA microarray.	A gene-expression profile of 35 genes, including CTSB, can predict the outcome of thermoradiotherapy for SCC.	[35]
				ELISA and qPCR was used to measure cathepsin B expression in HeLa cells and 169 tissue samples from invasive carcinomas, precancerous and normal tissues (*p <* 0.01).	Cathepsin B expression in invasive carcinomas was significantly higher as compared to precancerous tissue and normal tissue. Cathepsin B expression in the invasive carcinomas was positively correlated to tumor invasion depth and lymphatic metastasis. Significant regression of HeLa tumor growth in nude mice, which received HeLa cells treated with siRNA for CTSB.	[36]
6.	GSN	GELS_HUMAN	Gelsolin	Differential proteomics of HeLa cells *versus* non-tumorigenic cell line HaCaT	Gelsolin is overexpressed in HeLa cells as compared to HaCaT	[26]
				Differential expression of genes in tumor tissues and adjacent noncancerous mucosacancerous tissues was studied by microarray. Confirmation by Western blot and IHC. Plasma gelsolin measured by ELISA. HeLa gelsolin knockdown.	Gelsolin levels were significantly upregulated in 78% of patients with cervical cancer. Levels were higher in cervical tumor tissues than in the surrounding noncancerous tissues. Gelsolin abundance in the plasma of cervical cancer patients was increased 2.2-fold compared to healthy controls and was significantly different in the early and late stages. Survival and recurrence-free survival rates were significantly higher for the low-expression group. Cancer cells with reduced gelsolin expression exhibited reduced migration and proliferation.	[37]
				Plasma of patients with different grades of SCC	Correlation of serum gelsolin expression with CIN3 and different stages of SCC	[38]
7.	HMGB2	HMGB2_HUMAN	High mobility group protein B2	Microarray for identification of differentially expressed genes between untreated and TNF-treated cells. Confirmation by RT-PCR.	TNF treatment upregulated HMGB2 in E7-expressing cells but not in E7 negative cells.	[39]
8.	IL18	IL18_HUMAN	Interleukin-18	Secretome of cervical cancer cell lines SiHa and CaSki were investigated by ELISA.	E6 and E7 reside in the extracellular fluid of HPV-containing cervical cancer cell lines and inhibit IL-18-induced IFN-gamma production locally in HPV lesions through inhibition of IL-18 binding to its alpha-chain receptor. This effect does not occur with the IL-18 mutant E42A.	[40,41]
				HaCaT and C-33A, (HPV-negative cervical cancer cell line) stabile expressing E6, E6 mutant, E6E7, or E7 were investigated by RT-PCR.	E6 downregulated IL-18 mRNA expression, independent of p53 degradation, in HaCaT cells expressing a mutated p53 form.	[42]
				TaqMan Allelic Discrimination Assay was used to genotype IL-18 polymorphisms for women with SCC and healthy control women.	IL-18 1297 T/C, 607 C/A, 380 C/G, 137 G/C, and +105 A/C polymorphisms are not associated with susceptibility to SCC in Taiwanese women.	[43]
9.	MIF	MIF_HUMAN	Macrophage migration inhibitory factor	MIF protein and mRNA expression was analyzed by Western blotting, ELISA and RT-PCR in uterine cervical cancer cell lines SiHa and CaSki and their supernatant, and on 80 biopsies (cervical dysplasias, *in situ* carcinomas and invasive carcinomas) of uterine cervical tissue.	MIF is overexpressed in invasive cervical cancer as compared to cervical dysplasias. MIF is overexpressed in SiHA and CaSki cells and these cells also secrete the protein.	[44]
				250 patients with cervical cancer (49 cases with and 201 cases without lymphatic metastasis) were analyzed by RFLP and their serum by ELISA	Patients with GC and CC genotypes and C allele exhibited a lower degree of differentiation and a higher degree of malignancy. Early cervical cancer, lymphatic metastasis and poorly differentiated carcinomas exhibited higher MIF levels in serum. Moreover, patients with the CC genotype exhibited higher MIF serum concentration, which could increase the risk of early stage cervical cancer and lymphatic metastasis.	[45]
				Differential proteomics of HeLa cells *versus* non-tumorigenic cell line HaCaT	MIF is overexpressed in HeLa cells as compared to HaCaT	[26]
				MIF knockdown in HeLa cells	MIF is crucial for proliferation and tumorigenesis of human HeLa cells	[46]
				IHC on 209 tissue samples (40 normal cervical epithelia, 43 CIN 1, 41 CIN 2 to 3, and 85 SCC) and on cervical cancer cell lines SiHa and C-33A. Semi-quantitative PCR and Western blot on SiHa and C-33A	Overexpression of MIF in SCC and its precancerous lesions (CIN1-3) and in SiHa and C-33A indicates that MIF may play an important role in the pathogenesis of cervical cancer.	[47]
10.	MUC5B	MUC5B_HUMAN	Mucin 5B	Slot blot on normal and malignant cervical tissues	Increased expression of MUC5B in endometrial tumors but not in cervical tumors.	[48]
11.	MYL6	MYL6_HUMAN	myosin light chain 6	Subtractive hybridization and semi-quantitative PCR on 48A9 cells (subclone from Caski cells that were cultivated during space flight and had lower tumorigenic potential) and Caski cells.	MYL6 expression was >3-fold increased in cells exposed to spaceflight and with lower tumorigenic potential.	[49]
12.	OLFM4	OLFM4_HUMAN		OLFM4 expression and distribution was tested by IHC and RT-PCR on cervical intraepithelial neoplasia (CIN) and invasive SCC.	OLFM4 expression correlated with progression of CIN and differentiation of cervical cancer	[50]
13.	PDIA3	PDIA3_HUMAN	Protein disulfide-isomerase A3	Differential 2D-PAGE proteomics on paired adjacent normal and tumor tissues from 4 patients, comprising 2 SCC in stage IB1 and IIA, one adenocarcinoma (AD) in stage IB2, and one adenosquamous cell carcinoma in stage IB1. Western blot, IHC and shRNA konckdown were performed to confirm the results and to estimate clinical significance.	PDIA3 was overexpressed in 73% of cancers. PDIA3 expression was significantly higher in patients with AD compared with SCC. Staining was intense in AD with a penetration depth greater than half of the cervical stroma. High expression was associated with low overall survival and recurrence-free survival (RFS) rates. Patients exhibiting both high PDIA3 expression and lymph node metastasis displayed poorer outcomes than other patient groups. Knockdown of PDIA3 in HeLa cells decreased cell invasiveness and inhibited lung metastasis in a xenograft mouse model.	[51]
				Differential proteomics of HeLa cells *versus* non-tumorigenic cell line HaCaT	Protein Disulfide isomerase A3 is overexpressed in HeLa cells as compared to HaCaT	[26]
14.	PGAM1	PGAM1_HUMAN	Phosphoglycerate mutase 1	Differential 2D-PAGE proteomics on HeLa cells treated or not with suberonylanilide hydroxamic acid (SAHA). Confirmation on HeLa and CaSki cells by Western blot.	PGAM1 was significantly downregulated in HeLa and CaSki cells after treatment with HDAC1 inhibitor and antitumor agent SAHA.	[52]
				Differential proteomics of HeLa cells *versus* non-tumorigenic cell line HaCaT	Differential proteomics study shows that Phosphoglycerate mutase 1 is overexpressed in HeLa cells as compared to HaCaT	[26]
15.	S100P	S100P_HUMAN	Protein S100-P	Differential-display PCR for identification of differentially expressed mRNAs in cells containing inducible E7. Verification of S100P mRNA and protein expression by Northern and Western blot, respectively.	S100P mRNA and protein expression was down-regulated in the E7-expressing cells.	[53]
				HeLa, CGL3 and SiHa carcinoma cells as well as HCE16/3 immortalized cells were investigated for S100P expression. DNA methylation was inhibited by 5-aza-dC in S100P-negative cell lines CGL1 and Caski and the SP100 expressing SiHa cells. Suppression subtractive PCR between two HeLa cell lines with different tumerigenic capacity.	Expression of S100P gene in cervical carcinoma cells is not regulated by E7. S100P correlates with and contributes to the tumorigenic capacity of HeLa cells. Inhibition of DNA methylation resulted in induced S100P transcription in CGL1 and Caski but did not change S100P expression in SiHa cells. Suppression subtractive PCR identified differentially expressed genes, including S100P, with possible relevance for control of tumorigenic potential using two cervical carcinoma cell lines of the common HeLa origin, but of different capacity to generate tumors in nude mice.	[54,55]
16.	SERPINB13	SPB13_HUMAN	SERPINB13 protein	Microarray analysis on primary cervical cancer samples with or without lymph node metastasis and confirmation by semi-quantitative PCR.	SERPINB13 was involved in a set of proteins that could be used for prediction of lymph node metastasis.	[56]
17.	SOD1	SODC_HUMAN	Superoxide dismutase [Cu-Zn]	siRNA knowkdown and RT-PCR of Cervical cancer cell line SiHa.	Knockdown of SOD1 expression in SiHa cells significantly enhanced lipid peroxidation and cytotoxicity on exposure to docosahexaenoic acid	[57]
18.	SOD2	SODM_HUMAN	Superoxide dismutase 2	qPCR on SCC tissue from patients with or without lymph node metastasis	Correlation of SOD2 with lymph node metastasis in patients with early stage cervical carcinoma	[58]
				Microarray on HPV16/18 immortalized keratinocytes and confirmation by Northern blot. Immunohistochemistry on 331 cervical histological samples.	Correlation of SOD2 expression with differential NF-kB activation by TNF in HPV16/18 immortalized keratinocytes. Abnormal expression of SOD 2 correlates with different stages of cervical neoplasia.	[59,60]
19.	YWHAQ	1433T_HUMAN	14-3-3 protein theta	Differential proteomics on normal *versus* cervical SCC tissues.	Decreased abundance of 14-3-3 epsilon in SCC tissues	[28]
20.	YWHAE	1433E_HUMAN	14-3-3 protein epsilon	Immunostaining on 297 SCC tissues	Reduced immunostaining of 14-3-3 sigma protein in the cytoplasm and shuttle of 14-3-3 sigma protein into the nucleus may be two different mechanisms that determine the carcinogenesis of SCC	[61]
				qPCR on SCC tissue from patients with or without lymph node metastasis	Correlation of 14-3-3 zeta protein expression with lymph node metastasis in patients with early stage cervical carcinoma	[58]
				Proteomics of HeLa cells *versus* non-tumorigenic cell line HaCaT, verification by Western Blot and Cytoscape pathway analysis.	Differential proteomics study and subsequent pathway analysis reveals that 14-3-3 zeta protein is key in the determination towards proliferation or cell death. The 14-3-3 family members emerge as important regulators in carcinogenesis and as possible clinical targets.	[26,27]
				Pull-down in HeLa cells	E6 oncoproteins from HR-HPVs interact with 14-3-3 zeta protein via PDZ domain	[62]

#### 3.3.2. Only CVF from Precancerous Patients Contains Proteins That Make up the Central Core of Cervical Cancer

Recently, Higareda-Almarez *et al*. [26,27] compared the intracellular proteomes of six cervical cancer cell lines with the non-tumorigenic cell line HaCat and identified a consensus set of 66 unique or overexpressed cervical cancer proteins, which they called the “central core of cervical cancer”. As expected, many of these proteins had functions related to cancer hallmark processes such as cell migration/metastasis, evasion of apoptosis and central metabolism. In this network, the 14-3-3ζ scaffold protein featured as a highly interconnected hub, overexpressed in cervical cancer cells.

Cathepsin G, gelsolin, protein disulfide-isomerase A3, phosphoglycerate mutase 1, and annexin A4 were found to overlap between the list of 52 CVF ‘cancer pathway proteins’ that were unique for the precancerous condition and the list of Higareda-Almaraz *et al*. [26,27] (Table 3). Although 14-3-3ζ was not detected in our approach, the isoforms 14-3-3ε and 14-3-3θ were identified. Many studies have highlighted the importance of the 14-3-3 protein family in processes that are crucial for normal growth and development and that often become deregulated in human cancer (for an overview, see [63,64]). No overlap was observed with the 57 CVF unique ‘cancer pathway proteins’ from healthy individuals (Figure 1).

## 4. Conclusions

Shotgun proteomics often provides us with proteins that have a qualitative differential appearance in samples from healthy and diseased individuals. Since many of these proteins may belong to common pathways responsible for the disease, their interconnection and clustering will confirm their involvement and may tell us in which combinations they optimally act as biomarkers for a specific and sensitive bioassay. From the lists of unique CVF proteins we identified from healthy and precancerous women, pathway reconstruction was performed using the Ingenuity Pathway Analysis software.

Proteins in the CVF proteome from precancerous individuals tended to be more involved in pathways that contribute in cancer processes, when compared to CVF proteins from healthy individuals. Moreover, examination of the literature from CVF proteins categorized by IPA as being involved in ‘cancer processes’ showed that substantially more of these proteins correlated to some extent with cervical cancer when they originated from precancerous individuals (8/52 (15%, healthy) *versus* 19/57 (33%, precancerous)) and many of these were involved in a small sub-network. Thus, the general characteristics of both proteomes (functional distribution, number of proteins) did not differ markedly but when focusing on cancer features, clear differences could be observed (Figure 1). Altogether, these results reinforce the idea that parts of the cancer pathways within the precancerous cervical tissue are present in the CVF and such pathway biomarkers could, possibly in combination, very well be used as more reliable biomarkers for the diagnosis and prognosis of cervical cancer.

Frequently used targeting techniques for accurate quantification of a specific protein in clinical samples are nowadays ELISA and MRM [65,66,67]. ELISA offers the advantage of measuring in a (semi)-high throughput fashion whereby hundreds of samples can be tested at a reasonable time (days). However, highly specific antibodies are required to minimize background from the matrix. Indeed, in our hands, about half of the ELISAs we tested on CVF were unsatisfactory. MRM is a mass spectrometric technique, whereby proteotypic peptides, representative for the protein of interest, are selected in a first analyzer and fragmented in a collision cell as to detect a given fragment in a third analyzer. Quantification is possible after spiking of the sample with a known amount of proteotypic peptide containing heavy isotopes, enabling the mass spectrometer to distinguish between the endogenous and exogenous proteotypic peptides. As prior chromatography is usually required, MRM is more time consuming but several markers can be tested in one run.

Using these techniques, a set of proteins could be defined from CVF, preferably involved in different cancer pathways, allowing for sensitive and specific diagnosis of cervix cancer and its precancerous states. Since CVF is a body fluid that is easy to collect with the aid of an appropriate device [68], it opens up the possibility for development of a dipstick assay optimized for detection of the appropriate set of proteins. Such an assay could possibly be carried out by the woman herself, making it an appropriate test in countries where biomedical centers are difficult to reach due to geographical, financial, or other reasons. A first dipstick assay for cervical cancer screening, based on the detection of the viral E6 protein, was recently tested in a large cohort of Chinese women [69]. In this case, the samples were swabs taken by a local practitioner and measurement required additional manipulations (such as lysing the cells), thereby hampering further development towards a self-test. Nevertheless, specificity and positive predictive value of this E6 assay showed promising results although sensitivity remained relatively low, possibly because E6 protein levels were only clearly detectable at the later CIN stages 2 and 3. Once more, these results suggest that an easy applicable and reliable cervical cancer-screening test may become reality but requires more than one biomarker. Moreover, if carefully chosen, the gradual appearance of certain proteins (or combinations thereof) may tell us how far the tissue is evolved towards a carcinoma and/or whether clearing of the precancerous tissue will occur, all this from a body fluid that is easy to collect.

## Supplementary Materials

Supplementary File 1
